# Assessment of Pediatric Admissions for Kawasaki Disease or Infectious Disease During the COVID-19 State of Emergency in Japan

**DOI:** 10.1001/jamanetworkopen.2021.4475

**Published:** 2021-04-06

**Authors:** Takuya Hara, Kenji Furuno, Kenichiro Yamamura, Junji Kishimoto, Yumi Mizuno, Kenji Murata, Sagano Onoyama, Ken Hatae, Megumi Takemoto, Yoshito Ishizaki, Shunsuke Kanno, Kazuo Sato, Yoshitomo Motomura, Yasunari Sakai, Shouichi Ohga, Mayumi Yashiro, Yoshikazu Nakamura, Toshiro Hara

**Affiliations:** 1 Kawasaki Disease Center, Fukuoka Children’s Hospital, Kashiiteriha, Higashi-ku, Fukuoka, Japan; 2 Department of Perinatal and Pediatric Medicine, Graduate School of Medical Sciences, Kyushu University, Maidashi, Higashi-ku, Fukuoka, Japan; 3 Department of Research and Development of Next Generation Medicine, Faculty of Medical Sciences, Kyushu University, Maidashi, Higashi-ku, Fukuoka, Japan; 4 Division of Pediatrics, Japanese Red Cross Fukuoka Hospital, Ookusu, Minami-Ku, Fukuoka, Japan; 5 Division of Pediatrics, Hamanomachi Hospital, Nagahama, Chuo-ku, Fukuoka, Japan; 6 Division of Pediatrics, National Hospital Organization Fukuokahigashi Medical Center, Chidori, Koga, Fukuoka, Japan; 7 Division of Pediatrics, National Hospital Organization Kyushu Medical Center, Jigyohama, Chuo-ku, Fukuoka, Japan; 8 Department of Pediatrics, Graduate School of Medical Sciences, Kyushu University, Maidashi, Higashi-ku, Fukuoka, Japan; 9 Department of Public Health, Jichi Medical University, Yakushiji, Shimotsuke, Tochigi, Japan

## Abstract

**Importance:**

The development of Kawasaki disease (KD) has been suggested to be associated with droplet- or contact-transmitted infection; however, its triggers and transmission modes remain to be determined. Under an epidemic of SARS-CoV-2, the COVID-19 state of emergency in Japan served as a nationwide social experiment to investigate the impact of quarantine or isolation on the incidence of KD.

**Objective:**

To assess the role of droplet or contact transmission in the etiopathogenesis of KD.

**Design, Setting, and Participants:**

This multicenter, longitudinal, cross-sectional study was conducted from 2015 to 2020 at Fukuoka Children’s Hospital and 5 adjacent general hospitals. The number of admissions for KD and infectious diseases were analyzed. Participants were pediatric patients admitted to the participating hospitals for KD or infectious diseases.

**Exposures:**

Quarantine and isolation owing to the COVID-19 state of emergency.

**Main Outcomes and Measures:**

The primary end points were the ratios of patients with KD to patients with respiratory tract or gastrointestinal infections admitted from April to May in 2015 to 2019 and 2020. A Poisson regression model was used to analyze them.

**Results:**

The study participants included 1649 patients with KD (median [interquartile range] age, 25 [13-43] months; 901 boys [54.6%]) and 15 586 patients with infectious disease (data on age and sex were not available for these patients). The number of admissions for KD showed no significant change between April and May in 2015 to 2019 vs the same months in 2020 (mean [SD], 24.8 [5.6] vs 18.0 [4.0] admissions per month; 27.4% decrease; adjusted incidence rate ratio [aIRR], 0.73; 95% CI, 0.48-1.10; *P* = .12). However, the number of admissions for droplet-transmitted or contact-transmitted respiratory tract infections (mean [SD], 157.6 [14.4] vs 39.0 [15.0] admissions per month; 75.3% decrease; aIRR, 0.25; 95% CI, 0.17-0.35; *P* < .001) and gastrointestinal infections (mean [SD], 43.8 [12.9] vs 6.0 [2.0] admissions per month; 86.3% decrease; aIRR, 0.14; 95% CI, 0.04-0.43; *P* < .001) showed significant decreases between April and May in 2015 to 2019 vs the same months in 2020 (total, 12 254 infections). Thus, the ratio of KD to droplet- or contact-transmitted respiratory tract and gastrointestinal infections incidence in April and May 2020 was significantly increased (ratio, 0.40 vs 0.12; χ^2^_1_ = 22.76; *P* < .001).

**Conclusions and Relevance:**

In this study, the significantly increased incidence of KD compared with respiratory tract and gastrointestinal infections during the COVID-19 state of emergency suggests that contact or droplet transmission is not a major route for KD development and that KD may be associated with airborne infections in most cases.

## Key Points

**Question:**

Is Kawasaki disease (KD) associated with droplet- or contact-transmitted infection?

**Findings:**

In this cross-sectional study of 17 235 pediatric patients, the number of admissions for KD showed no significant change (27.4% decrease) during quarantine owing to the COVID-19 pandemic, whereas there were significant decreases in numbers of hospital admissions for droplet-transmitted or contact-transmitted respiratory tract infections (75.3% decrease) and gastrointestinal infections (86.3% decrease). Thus, the ratio of KD admissions to admissions for these infections increased.

**Meaning:**

These findings suggest that contact or droplet transmission is not a major route for KD development and that KD may be associated with airborne disease.

## Teaser

This cross-sectional study assesses the role of droplet or contact transmission in the etiopathogenesis of Kawasaki disease.

## Introduction

Kawasaki disease (KD) is an acute, self-limited, febrile disease that predominantly affects children aged 6 months to 5 years and is characterized by systemic small and medium vessel vasculitis.^1,2,3^ Several lines of evidence suggest that KD occurs in genetically predisposed patients after exposure to certain triggers in the surrounding environment.^2,3,4^ There is consistent evidence for genetic susceptibility. Epidemiological features, such as increased prevalence among siblings and twins^5,6^ and distinct prevalence rates between ethnic groups regardless of residence,^7,8,9^ are well known. Furthermore, KD susceptibility genes, including *ITPKC*, *ORAI1*, *CASP3*, *BLK*, *CD40*, and *FCGR2A*, have been identified.^10,11^

Despite long-term active research, the triggers for KD remain unknown. One dominant theory is that KD arises after infection, particularly by pathogens transmitted via contact or droplets.^12,13,14^ However, controversy remains regarding whether the major trigger is infectious, whether it is single or multiple, and how the transmission occurs.

Since the end of 2019, SARS-CoV-2, the causative agent of COVID-19, has spread rapidly throughout the world. In Japan, the first SARS-CoV-2 outbreaks occurred in February 2020.^15^ Under these conditions, handwashing, masks, and physical distancing were urged, and the government declared a COVID-19 state of emergency, which lasted from April 7 to May 25, 2020. Consequently, the chances of exposure to not only SARS-CoV-2 but also other pathogens that can be spread by contact or droplets were decreased. To our knowledge, no data are currently available for the preventive effect of the nationwide quarantine on the occurrence of KD during the COVID-19 pandemic.

In this cross-sectional study, we aimed to clarify whether pathogens spread by contact or droplets were associated with KD development in Fukuoka, Japan, during the COVID-19 state of emergency owing to the SARS-CoV-2 epidemic. For this, we analyzed the incidence of KD in comparison with the incidence of infections, specifically respiratory tract and gastrointestinal infections, because most of these are transmitted by droplets or contact. We also investigated the changes in clinical features and presence of SARS-CoV-2 in patients with KD in 2020.

## Methods

The ethical committees of all participating hospitals approved the research protocol. Informed consent for parents was substituted with an opt-out process, given the retrospective nature of this study in the ethical committees’ approval. This report adheres to the Strengthening the Reporting of Observational Studies in Epidemiology (STROBE) reporting guideline.

This multicenter, longitudinal, cross-sectional study was designated the Quarantine and Social Isolation for COVID-19 and its Impact on Kawasaki Disease Study. We investigated the incidence of KD and infectious diseases during and after the COVID-19 state of emergency, when handwashing, masks, and physical distancing were urged, in Fukuoka Children’s Hospital and 5 adjacent general hospitals (details are shown in eAppendix 1 and eFigure 1 in the Supplement). The primary end point was the ratios of the number of patients with KD to the number of patients with respiratory tract or gastrointestinal infections admitted from April to May in 2015 to 2019 and from April to May in 2020. The secondary end point was the clinical features of KD in 2020.

### Statistical Analysis

The incidence rates of KD and infectious diseases were analyzed using a Poisson regression model. Continuous variables were tested for normalcy using a Shapiro-Wilk test and were compared between groups using an unpaired *t *test or the Mann-Whitney *U* test, as appropriate. Categorical variables were compared using the Fisher exact test. A 2-tailed *P* < .05 was chosen as the cutoff for significance. Statistical analyses were performed using R statistical software version 3.6.3 (The R Project for Statistical Computing) and JMP Pro statistical software version 15 software (SAS Institute). Additional details of the data collection and statistical analysis are described in eAppendix 2 and eAppendix 3 in the Supplement.

## Results

### Numbers of Patients With KD Before, During, and After the COVID-19 State of Emergency in 2020

**Figure 1.**
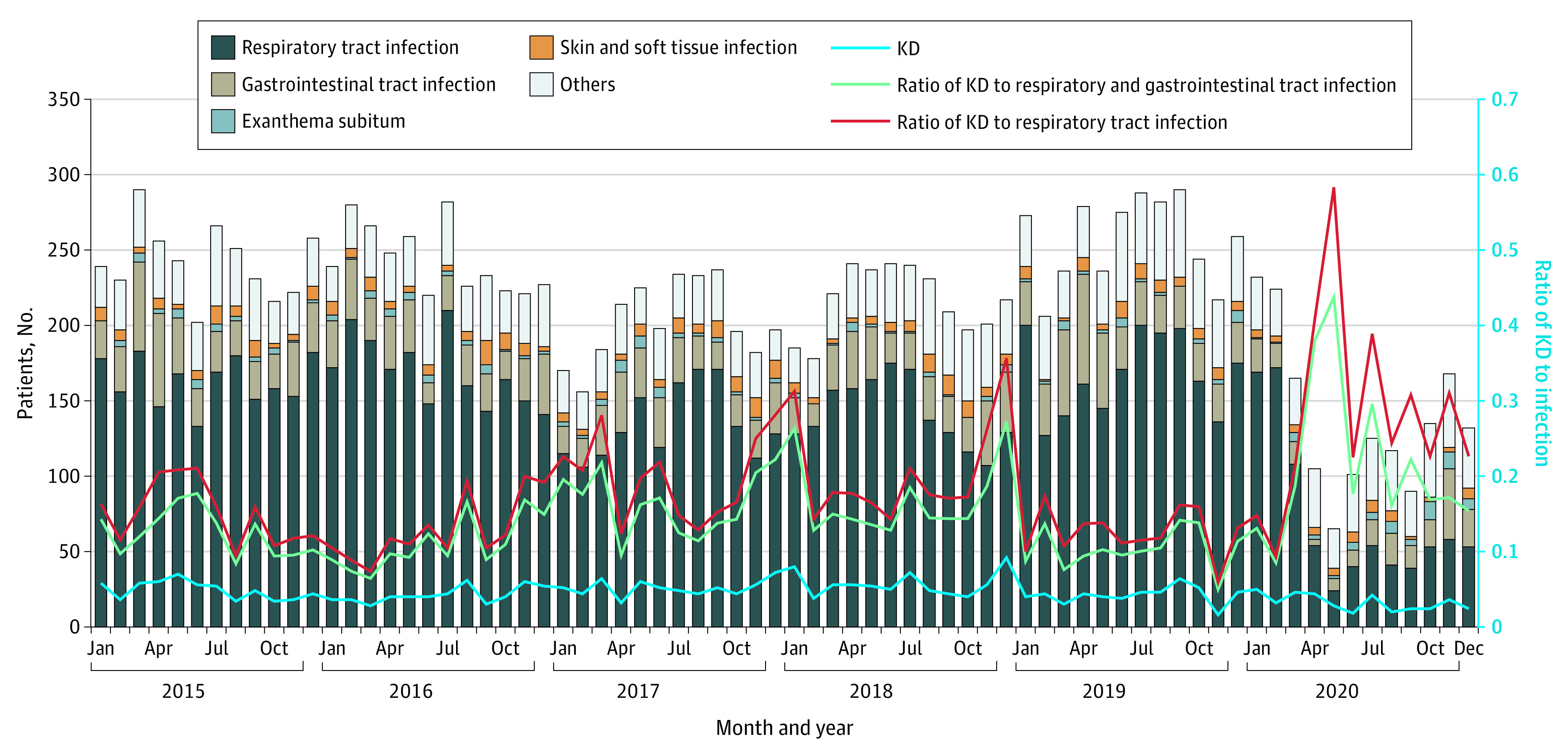
Ratios of the Numbers of Patients With Kawasaki Disease (KD) to Those of Patients With Either Respiratory Tract or Gastrointestinal Infections or to Those of Patients With Respiratory Tract Infection Across 6 Hospitals in Fukuoka

A total of 1649 patients with KD (median [interquartile range {IQR}] age, 25 [13-43] months; 901 boys [54.6%]) were admitted to 1 of 6 hospitals in Fukuoka during the period from 2015 to 2020. Compared with the numbers of admissions in April and May 2015 to 2019, the numbers of admissions for KD in April and May 2020 showed a 27.4% decrease, but this difference did not reach statistical significance (mean [SD], 24.8 [5.6] vs 18.0 [4.0] admissions per month; adjusted incidence rate ratio [aIRR], 0.73; 95% CI, 0.48-1.10; *P* = .12) (Table 1). To evaluate the change after the COVID-19 state of emergency, differences in the admissions for KD in June to December between 2020 and 2015 to 2019 were assessed. The number of patients with KD in June to December 2020 was significantly lower compared with the same period in 2015 to 2019 (mean [SD], 13.4 [4.1] vs 24.5 [6.7] admissions per month; 45.1% decrease; aIRR, 0.55; 95% CI, 0.41-0.73; *P* < .001) (Figure 1 and Table 1).

**Table 1.** IRRs in the Analysis of KD and Infectious Diseases

Disease	April to May 2020 vs 2015-2019				June to December 2020 vs 2015-2019			
	Hospital admissions/mo, mean (SD), No.		Adjusted IRR (95% CI)	*P* value	Hospital admissions/mo, mean (SD), No.		Adjusted IRR (95% CI)	*P* value
	2020	2015-2019^a^			2020	2015-2019^a^		
KD								
6 Hospitals	18.0 (4.0)	24.8 (5.6)	0.73 (0.48-1.10)	.12	13.4 (4.1)	24.5 (6.7)	0.55 (0.41-0.73)	<.001
11 Hospitals	41.0 (9.0)	51.5 (4.4)	0.88 (0.66-1.19)	.40	25.3 (2.7)	47.8 (8.2)	0.58 (0.47-0.73)	<.001
Infectious diseases (6 hospitals)								
Total infectious diseases	85.0 (20.0)	243.8 (17.3)	0.35 (0.28-0.49)	<.001	124.0 (23.6)	232.5 (27.7)	0.53 (0.47-0.61)	<.001
Respiratory infections	39.0 (15.0)	157.6 (14.4)	0.25 (0.17-0.35)	<.001	48.3 (7.4)	155.4 (25.8)	0.31 (0.25-0.39)	<.001
Gastrointestinal infections	6.0 (2.0)	43.8 (12.9)	0.14 (0.04-0.43)	<.001	22.0 (11.0)	26.9 (6.2)	0.82 (0.64-1.05)	.12
Exanthema subitum	2.5 (0.5)	4.7 (2.2)	0.53 (0.20-1.39)	.16	7.4 (2.9)	3.0 (1.9)	2.45 (1.69-3.55)	<.001
Skin and soft tissue infections	5.0 (0.0)	5.2 (2.0)	0.96 (0.53-1.74)	.90	5.3 (2.3)	8.3 (3.1)	0.63 (0.44-0.92)	.01
Representative infectious diseases with a pathogen identified (6 hospitals)								
RSV	4.0 (4.0)	18.7 (5.7)	0.21 (0.07-0.66)	<.001	NA	NA	NA	NA
Rotavirus	0.0 (0.0)	19.3 (11.0)	NC^b^	NC^b^	NA	NA	NA	NA
Infectious diseases nationwide (data from NESID)								
RSV	0.17 (0.13)^c^	1.11 (0.46)	0.12 (0.03-0.47)	.002	0.27 (0.18)	4.50 (3.26)	0.06 (0.008-0.40)	<.001
Rotavirus	0.04 (0.02)	2.31 (0.67)	0.02 (0.0007-0.41)	<.001	0.004 (0.005)	0.19 (0.25)	0.09 (0.002-3.13)	.04

Abbreviations: IRR, incidence rate ratio; KD, Kawasaki disease; NA, not available; NC, not calculable; NESID, National Epidemiological Surveillance for Infectious Diseases; RSV, respiratory syncytial virus.

^a^ Data for 2015 to 2019 are for 6 hospitals, and data for 2017 to 2019 are for 11 hospitals and the data from NESID.

^b^ IRR, 95% CI, and adjusted *P* value of rotavirus in April to May 2020 were not calculable because the number of cases was 0.

^c^ Data from NESID are shown as the mean number of patients per sentinel site per month.

**Figure 2.**
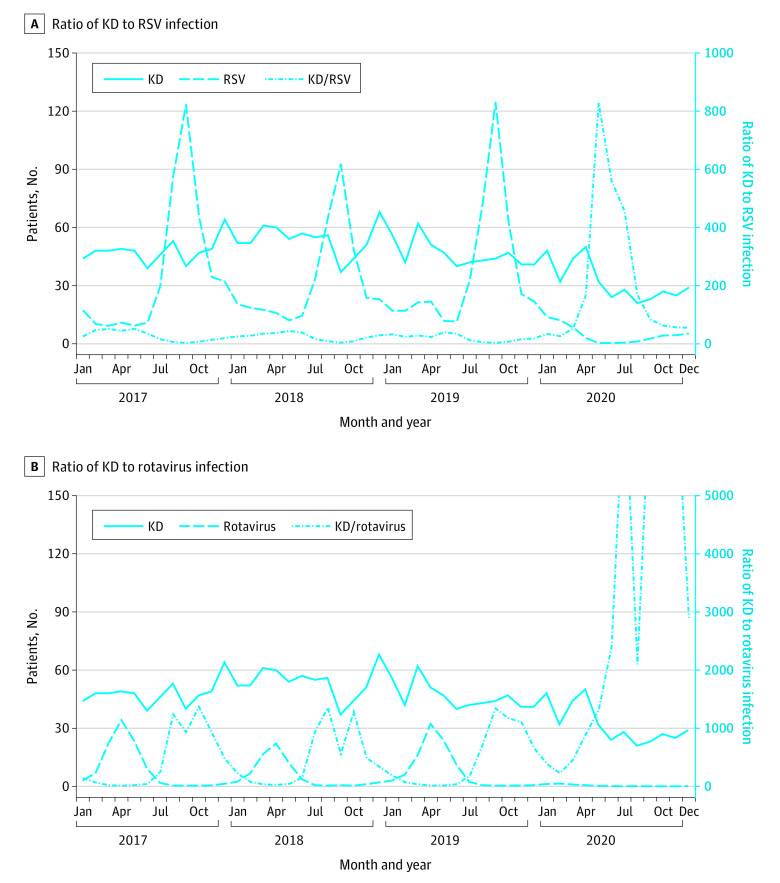
Ratios of the Numbers of Patients With Kawasaki Disease (KD) to Those of Patients With Respiratory Syncytial Virus (RSV) or Rotavirus Infection Across 11 Hospitals Nationwide Panel A shows the ratio of KD to RSV infection, and panel B shows the ratio of KD to rotavirus infection. Numbers of patients with KD across 11 hospitals were derived from the KD Rapid Report System. Numbers of patients with RSV and rotavirus infections per sentinel site were derived from the National Epidemiological Surveillance for Infectious Diseases in Japan. The mean number of patients with RSV or rotavirus is displayed as the total number multiplied by 10 per sentinel site.

To investigate the change in the number of KD admissions in Japan, we used data for 2161 admissions from 11 hospitals in other areas of Japan (KD Rapid Report System) from which reliable year-round data were available for 2017 to 2020 (Figure 2). The number of patients with KD admitted to the 11 hospitals in April to May 2020 showed a 20.4% decrease from the same months in 2017 to 2019, although this difference did not reach significance (mean [SD], 41.0 [9.0] vs 51.5 [4.4] admissions per month; aIRR, 0.88; 95% CI, 0.66-1.19; *P* = .40) (Table 1). The number of patients with KD after the COVID-19 state of emergency (June to December 2020) showed a significant decrease compared with the same period in 2017 to 2019 (mean [SD], 25.3 [2.7] vs 47.8 [8.2] admissions per month; 47.1% decrease; aIRR, 0.58; 95% CI, 0.47-0.73; *P* < .001) (Figure 2 and Table 1), just like in our 6 hospitals.

### Numbers of Patients With Infectious Diseases Admitted to the 6 Hospitals in 2020 Compared With Those in 2015 to 2019

From 2015 to 2020, 15 586 patients with infectious diseases were admitted to the 6 participating hospitals in Fukuoka (data on age and sex were not available for these patients). In April to May 2020, the numbers of total infectious disease cases declined compared with the same period in 2015 to 2019 (mean [SD], 85.0 [20.0] vs 243.8 [17.3] admissions per month; 65.1% decrease; aIRR, 0.35; 95% CI, 0.28-0.49; *P* < .001) (Figure 1 and Table 1). Specifically, the numbers of patients with respiratory tract or gastrointestinal infections (total, 12 254 infections), which are mainly spread by droplets or contact, presented significant decreases (respiratory tract infections, mean [SD], 157.6 [14.4] vs 39.0 [15.0] admissions per month; 75.3% decrease; aIRR, 0.25; 95% CI, 0.17-0.35; *P* < .001; gastrointestinal infections, mean [SD], 43.8 [12.9] vs 6.0 [2.0] admissions per month; 86.3% decrease; aIRR, 0.14; 95% CI, 0.04-0.43; *P* < .001) (Figure 1 and Table 1). After the COVID-19 state of emergency was lifted, in June to December 2020, the number of patients with infectious disease remained lower compared with the same period in 2015 to 2019, although the difference for gastrointestinal infections was not significant (respiratory tract infections, mean [SD], 48.3 [7.4] vs 155.4 [25.8] admissions per month; 68.9% decrease; aIRR, 0.31; 95% CI, 0.25-0.39; *P* < .001; gastrointestinal infections, mean [SD], 22.0 [11.0] vs 26.9 [6.2] admissions per month; 18.2% decrease; aIRR, 0.82; 95% CI, 0.64-1.05; *P* = .12) (Figure 1 and Table 1).

The numbers of admissions for exanthema subitum and skin and soft-tissue infections did not show significant changes between April to May 2020 and April to May 2015 to 2019 (Table 1). However, in June to December 2020, the numbers of admissions for exanthema subitum significantly increased compared with June to December 2015 to 2019 (mean [SD], 7.4 [2.9] vs 3.0 [1.9] admissions per month; aIRR, 2.45; 95% CI, 1.69-3.55; *P* < .001), whereas those for skin and soft-tissue infections significantly decreased (mean [SD], 5.3 [2.3] vs 8.3 [3.1] admissions per month; aIRR, 0.63; 95% CI, 0.44-0.92; *P* = .01) (Table 1). In the analysis of identified pathogens for respiratory tract infections and gastrointestinal infections, the numbers of patients admitted with respiratory syncytial virus (RSV) (mean [SD], 4.0 [4.0] vs 18.7 [5.7] admissions per month; aIRR, 0.21; 95% CI, 0.07-0.66; *P* < .001), human metapneumovirus (1.5 [1.5] vs 17.8 [11.3] admissions per month), rotavirus (mean [SD], 0 [0] vs 19.3 [11.0] admissions per month), and norovirus (0 [0] vs 3.3 [3.0] admissions per month) (aIRR and 95% CI were not calculable for rotavirus and norovirus because the number of cases was 0) showed significant decreases during the period of strict social isolation in April to May 2020 compared with April to May 2015 to 2019 (Table 1). Influenza virus was difficult to assess because its off season was April to May in 2018 and 2020. The number of patients admitted with *Streptococcus pyogenes* was too small to obtain statistical significance (1.5 [0.5] vs 1.9 [1.0] admissions per month in April to May 2020 vs April to May 2015 to 2019).

### Ratios of Admissions for KD to Those for Respiratory Tract and Gastrointestinal Infections in 2015 to 2020

The trends in the monthly numbers of patients admitted with KD or an infectious disease to the 6 participating hospitals in Fukuoka are presented in Figure 1. To compare the changes in admission numbers between KD and respiratory tract or gastrointestinal infections, the ratios of admissions for KD to those for infectious diseases were analyzed. The ratio of KD to respiratory tract and gastrointestinal infections in April to May 2020 increased compared with the same period in 2015 to 2019 (0.40 vs 0.12; χ^2^_1_ = 22.76; *P* < .001) because of the large decrease in those infections with only a small decrease in KD (Figure 1). The number of patients with KD in June to December 2020 (ie, after the COVID-19 state of emergency) decreased by 45% in 6 Fukuoka hospitals and by 47% in 11 nationwide hospitals compared with the same period in 2015 to 2019. In contrast, the number of patients with respiratory tract infection continued to be very low even after the strict isolation period, and the ratio of KD to respiratory tract infection remained significantly higher in June to December 2020 compared with the same period in 2015 to 2019 (0.28 vs 0.16; χ^2^_1_ = 16.47; *P* = .004) (Figure 1).

To evaluate the role of droplet or contact transmission more precisely, we further analyzed the ratio of KD to specific respiratory tract or gastrointestinal infections before, during, and after the COVID-19 state of emergency. The ratio of the number of patients with KD to that of patients with RSV or rotavirus infection across the 6 hospitals increased (eFigure 2 in the Supplement). To investigate the nationwide change, we analyzed data from the KD Rapid Report System and National Epidemiological Surveillance for Infectious Diseases. The numbers of patients with RSV and rotavirus infections decreased to less than 6% in April to May and June to December 2020 (Table 1). Therefore, the ratio of the number of patients with KD to that of patients with RSV or rotavirus infection increased across 11 hospitals (Figure 2).

### Clinical Features of Patients With KD in 2020

The clinical features of patients with KD in April to May 2015 to 2019 and 2020 are presented in Table 2. Patients with KD in April to May 2020 had significantly lower white blood cell counts (median [IQR], 12 800 [9 600 to 15 600] cells/μL vs 14 800 [11 7 to 19 400] cells/μL; difference, −2300 cells/μL; 95% CI, −700 to −3900 cells/μL; *P* = .01) (to convert white blood cell counts to ×10^9^ cells/L, multiply by 0.001) and neutrophil percentages (median [IQR], 69.3% [50.4% to 76.0%] vs 72.3% [58.9% to 84.8%]; difference, −6.7%; 95% CI, −0.2% to −13.2%; *P* = .04) (to convert neutrophil percentage to proportion of 1.0, multiply by 0.01), and showed a significantly lower frequency of intravenous immunoglobulin resistance (3 patients [8.3%] vs 69 patients [27.8%]; odds ratio, 0.24; 95% CI, 0.07 to 0.79; *P* = .01) compared with those in April to May 2015 to 2019.

**Table 2.** Clinical Information of Patients With KD During April to May, in 2015 to 2019 and 2020

Characteristic	Patients, No. (%)		*P* value
	2020 (n = 36)	2015-2019 (n = 248)	
Age at onset, median (IQR), mo	22 (11-38)	29 (13-48)	.11^a^
Sex			
Female	14 (38.9)	109 (44.0)	.60^b^
Male	22 (61.1)	139 (56.0)	
Day of illness at diagnosis, mean (SD)	4.9 (2.2)	5.0 (2.0)	.73^c^
Laboratory values just before treatment			
White blood cell count, median (IQR), cells/μL	12 800 (9 600-15 600)	14 800 (11 700-19 400)	.01^a^
Neutrophil percentage, median (IQR), %^d^	69.3 (50.4-76.0)	72.3 (58.9-84.8)	.04^a^
Hemoglobin, median (IQR), g/dL	11.4 (10.8-12.2)	11.5 (10.8-12.2)	.75^a^
Platelet, median (IQR), ×10^3^/μL	326 (259-403)	331 (279-411)	.45^a^
Aspartate aminotransferase, median (IQR), U/L	38 (28-105)	33 (26-64)	.13^a^
Alanine aminotransferase, median (IQR), U/L	27 (13-86)	18 (12-73)	.25^a^
Serum sodium, median (IQR), mEq/L	135 (134-137)	135 (134-137)	.74^a^
Total bilirubin, median (IQR), mg/dL^e^	0.60 (0.40-1.05)	0.54 (0.40-0.89)	.55^a^
C-reactive protein, median (IQR), mg/dL	7.1 (3.8-12.7)	7.4 (4.4-11.9)	.84^a^
Risk score^f^			
Kobayashi score, median (IQR)	3 (1-5)	3 (1-5)	.86^a^
Egami score, median (IQR)	2 (1-3)	2 (1-2)	.45^a^
Osaka score, median (IQR)	1 (0-1)	1 (0-1)	.67^a^
Final diagnosis			
Incomplete KD	3 (8.3)	15 (6.0)	.49^b^
IVIG resistant	3 (8.3)	69 (27.8)	.01^b^
Cardiac complications			
Coronary artery dilatation before IVIG^g^	3 (8.3)	14 (5.6)	.46^b^
Cardiac complications at 4 weeks after onset	2 (5.6)	6 (2.4)	.27^b^

Abbreviations: IQR, interquartile range; IVIG, intravenous immunoglobulin; KD, Kawasaki disease.

SI conversion factors: To convert alanine aminotransferase to microkatals per liter, multiply by 0.0167; aspartate aminotransferase to microkatals per liter, multiply by 0.0167; C-reactive protein to milligrams per liter, multiply by 10.0; hemoglobin to grams per liter, multiply by 10.0; neutrophil percentage to proportion of 1.0, multiply by 0.01; platelets to ×10^9^ cells per liter, multiply by 1.0; serum sodium to millimoles per liter, multiply by 1.0; total bilirubin to micromoles per liter, multiply by 17.104; white blood cell count to ×10^9^ cells per liter, multiply by 0.001.

^a^ Calculated with Mann-Whitney *U* test.

^b^ Calculated with Fisher exact test.

^c^ Calculated with *t* test.

^d^ Data on neutrophil percentage were missing for 2 patients.

^e^ Data on total bilirubin were missing for 7 patients.

^f^ The Kobayashi score consists of age (1 point), day of illness at diagnosis (2 points), neutrophil percentage (2 points), platelet count (1 point), aspartate aminotransferase level (2 points), serum sodium level (2 points), and C-reactive protein level (1 point) with a score of 5 or higher indicating IVIG resistance. The Egami score consists of age (1 point), day of illness (1 point), alanine aminotransferase level (2 points), platelet count (1 point), and C-reactive protein level (1 point), with a score of 3 or higher indicating IVIG resistance. The Osaka score consists of aspartate aminotransferase level (1 point), total bilirubin level (1 point), and C-reactive protein level (1 point), with a score of 2 or higher indicating IVIG resistance.

^g^ One patient did not undergo an echocardiogram before treatment.

Among the 169 patients admitted for KD since February 2020, SARS-CoV-2 involvement was examined as follows: 68 patients were evaluated by polymerase chain reaction during the acute KD phase, 64 by a serological test at least 1 month from KD onset, and 37 by clinical and laboratory findings and family histories. No patients were found to be infected with SARS-CoV-2.

## Discussion

Here, we investigated the association of strict inhibition of contact and droplet infections under the COVID-19 stage of emergency with the incidence of KD during April to May 2020 (eFigure 3 in the Supplement). The numbers of patients with KD were decreased during April to May 2020 in the 6 hospitals in Fukuoka and 11 hospitals in other areas of Japan. Meanwhile, the number of hospitalizations for respiratory tract or gastrointestinal infections, such as RSV and rotavirus, decreased in April to May 2020 because they are mainly spread by droplets or contact. Therefore, the ratios of KD to respiratory tract and gastrointestinal infections in the 6 participating hospitals and the 11 nationwide hospitals in April to May 2020 were increased owing to the combination of pronounced decreases in the 2 infections and only a slight decrease in KD (Figure 1 and Figure 2 and eFigure 2 in the Supplement). These results suggest that transmission by contact or droplets is not a major route for KD spread and support the hypothesis that KD may be associated with airborne disease.

There is general agreement that KD occurs when genetically predisposed individuals are exposed to certain triggers.^2,3^ Various microbes spread by droplets or contact have been reported as associated with KD,^12,13,14^ but their causal effects remain to be confirmed in most cases.^16,17,18,19^ Only a few microbes have been recognized to be reproducibly associated with KD. In Japan, children infected with *Yersinia pseudotuberculosis* developed KD at rates of 12% to 35%.^20,21^ In Europe, KD incidence was found to increase with increasing risk of exposure to *Y pseudotuberculosis* infection.^22^ Furthermore, approximately 10% of patients hospitalized with KD in certain areas of Japan had serological evidence of *Y pseudotuberculosis* infection.^23,24^ Additionally, analysis by liquid chromatography–tandem mass spectrometry revealed possible pathogen-associated molecular patterns from *Y pseudotuberculosis* in KD serum samples.^25,26^ Nevertheless, an association with *Y pseudotuberculosis* can account for only less than 1% of the total KD cases in Fukuoka (authors’ unpublished data) and approximately 10% of total KD cases in Chugoku district in Japan.^23^

SARS-CoV-2 has been reproducibly associated with the development of KD and Kawasaki-like disease in Europe^27,28,29^ and the US,^30,31,32^ despite the low incidence rates (1 case per 300-6000 SARS-CoV-2 exposed children in Italy and the US).^33^ SARS-CoV-2 infects endothelial and immune cells via the angiotensin-converting enzyme 2 receptor.^34,35^ Elements of the virus were detected in endothelial cells, and endothelial inflammation was observed in patients with COVID-19.^36,37^ However, because KD onset in patients with COVID-19 typically occurs at 2 to 4 weeks after infection,^38,39^ KD is likely to be an immune-mediated disease rather than a direct consequence of the viral infection. To our knowledge, there have been no reports of unique KD patients associated with SARS-CoV-2 infection in Japan.

Recent studies^40,41,42,43,44,45^ have suggested that KD cases may be associated with local winds or large-scale wind currents that potentially carry airborne environmental triggers. Through liquid chromatography–mass spectrometry analyses of KD serum samples, common KD-associated molecules were simultaneously detected at several remote sites, despite exhibiting seasonal accumulation.^25,26^ The high ratios of KD to respiratory tract and gastrointestinal infections in April and May 2020 (Figure 1) suggest that airborne environmental triggers are associated with KD development for most cases.

Interestingly, there was a continued decrease in the number of patients with KD in June to December 2020 after the COVID-19 state of emergency, although the change was not comparable in scale to the decrease in the number of infectious diseases. In this period, the KD incidence did not decrease to less than half of that in the previous 5 years, whereas the incidence rates of contact- or droplet-transmittable RSV and rotavirus infections decreased to less than 6%. These persistently low RSV and rotavirus incidence rates suggest that people continued engaging in infection-preventive behaviors (eg, physical distancing, hand washing, and wearing masks) even after the COVID-19 state of emergency was lifted.

The present study has raised the following 2 possibilities for the persistence of KD decrease. First, airborne transmission associated with KD might be partly blocked by COVID-19 preventive behaviors, such as face masks and physical distancing. Epidemiological studies^46^ have revealed that patients with KD often have a history of upper respiratory infection before onset and that siblings or parents commonly experience coldlike symptoms before the development of KD in the family. Thus, masks may partially protect children from a possible airborne infection^47^ in a direct or indirect way.

Second, the quantity of a putative airborne environmental trigger capable of inducing KD might be diminished as a consequence of restricted socioeconomic activity. Decreases in the levels of environmental pollutants after some 2020 SARS-CoV-2 outbreaks, owing to restrictions on motor vehicles, power plants, and industrial facilities, have been reported.^48^ Given that microbes (viruses, bacteria, and fungi) can attach to air pollutants, including fine particulate matter (PM)^49^ with a diameter of less than 2.5 µm (PM_2.5_) and 0.1 µm (PM_0.1_), the decrease of KD incidence following the COVID-19 state of emergency might, at least in part, be associated with the reduction of a putative KD trigger. Although KD development is not associated with PM_2.5_,^50^ further study on microbes in PM_2.5_ or PM_0.1_ remains needed. Airborne triggers associated with KD must have weak or no pathogenicity to most children because only a small proportion of children develop KD after exposure. In addition, transmission by contact or droplets may also be involved in a small group of patients with KD.

The number of admissions for exanthema subitum, which is caused by transmission of human herpesviruses 6 and 7 from parent to child,^51,52^ did not decrease during April to May 2020 but increased in June to December 2020. The decrease might be associated with an increase of intrafamilial contact by more frequent working from home. In skin and soft-tissue infections, *Staphylococcus aureus*, including community-associated methicillin-resistant *S aureus*, was the most frequently identified pathogen. Therefore, airborne transmission^53^ in residential and community environments may be involved both in its continued occurrence during April to May 2020 and in the decrease in June to December 2020. This trend was similar to that for KD in these periods.

Regarding KD severity, the children admitted during April to May 2020 under the COVID-19 state of emergency showed milder symptoms compared with those admitted in 2015 to 2019. Because innate immunity plays a critical role in acute KD,^3,4^ the clinical features of KD may change in the absence of various infectious stimuli owing to the lack of hyperinflammation boosting via innate immune memory (ie, trained immunity).^54^

### Limitations

There are several limitations to the present study. First, this was a small, short-term study with limited generalizability, although we compared our findings with national data. Second, it was an epidemiological observational study, so it is difficult to directly speculate on the pathogenesis of KD. Despite these limitations, we believe that the results of this work provide intriguing clues toward clarification of the pathogenesis of KD and the establishment of preventive methods for KD.

## Conclusions

In this study, the ratio of KD to droplet- or contact-transmitted respiratory tract and gastrointestinal infections during the COVID-19 state of emergency in April to May 2020 was significantly increased because of the large decrease in the 2 infections and the smaller decrease in KD. Furthermore, the number of KD cases remained significantly lower from shortly after this period. These findings suggest that transmission by contact or droplets is not a major route for KD development in Japan and support the findings of previous epidemiological studies^40,41,42,43,44,45^ indicating that KD may be associated with airborne disease in most cases. More extensive studies are warranted for further understanding of this intriguing disease.

## Notes

Supplement. **eAppendix 1.** Data Source and Study Population **eAppendix 2.** Data Collection **eAppendix 3.** Statistical Analysis **eReference** **eFigure 1.** Geographical Locations of the FCH KD Center and Five Adjacent Hospitals, and the Number of Patients With KD **eFigure 2.** Ratios of the Number of Patients With KD to That of Patients With RSV or Rotavirus Infection Across Six Hospitals in Fukuoka **eFigure 3.** Timeline of COVID-19 Cases and Policies in Japan
