# Error in Discussion

**DOI:** 10.1001/jamanetworkopen.2021.14112

**Published:** 2021-05-26

In the Original Investigation titled “Assessment of Pediatric Admissions for Kawasaki Disease or Infectious Disease During the COVID-19 State of Emergency in Japan,”^1^ published April 6, 2021, there was an error in the second sentence of the eighth paragraph of the Discussion section. The word “decrease” should have appeared as “increase.” This article has been corrected.^1^
