# Supplementary material for: Assessment of Pediatric Admissions for Kawasaki Disease or Infectious Disease During the COVID-19 State of Emergency in Japan
Source: JAMA Netw Open. 2021 Apr 6;4(4):e214475. doi: 10.1001/jamanetworkopen.2021.4475 (PMC8025113; doi:10.1001/jamanetworkopen.2021.4475)
Supplement: Supplement. — eAppendix 1. Data Source and Study Population eAppendix 2. Data Collection eAppendix 3. Statistical Analysis eReference eFigure 1. Geographical Locations of the FCH KD Center and Five Adjacent Hospitals, and the Number of Patients With KD eFigure 2. Ratios of the Number of Patients With KD to That of Patients With RSV or Rotavirus Infection Across Six Hospitals in Fukuoka eFigure 3. Timeline of COVID-19 Cases and Policies in Japan [file jamanetwopen-e214475-s001.pdf]

Supplemental Online Content

Hara T, Furuno K, Yamamura K, et al. Assessment of pediatric admissions for Kawasaki disease or infectious disease during the COVID-19 state of emergency in Japan. *JAMA Netw Open*. 2021;4(4):e214475. doi:10.1001/jamanetworkopen.2021.4475

eAppendix 1. Data Source and Study Population
eAppendix 2. Data Collection
eAppendix 3. Statistical Analysis
eReference
eFigure 1. Geographical Locations of the FCH KD Center and Five Adjacent Hospitals, and the Number of Patients With KD
eFigure 2. Ratios of the Number of Patients With KD to That of Patients With RSV or Rotavirus Infection Across Six Hospitals in Fukuoka
eFigure 3. Timeline of COVID-19 Cases and Policies in Japan

This supplemental material has been provided by the authors to give readers additional information about their work.

© 2021 Hara T et al. *JAMA Network Open.*

eAppendix 1. Data Source and Study Population

Fukuoka Children's Hospital (FCH) and five adjacent general hospitals (Kyushu University Hospital, National Hospital Organization Kyushu Medical Center, Japanese Red Cross Fukuoka Hospital, Hamanomachi Hospital, and National Hospital Organization Fukuokahigashi Medical Center) participated in the study. These six hospitals comprise the major secondary or tertiary pediatric referral centers in Fukuoka; all five adjacent hospitals are located within 10 km of FCH (eFigure 1 in the supplement). To exclude any potential referral bias, we included admissions for KD and infectious diseases in five adjacent major hospitals. Because the "FCH alone" data did not differ significantly from the "FCH plus five adjacent hospitals" data, only the six-hospital data are presented in the Results section.

The inclusion criteria were: 1) patients aged 0–16 years; 2) patients admitted from 2015–2020; and 3) patients admitted with a diagnosis of KD or infectious disease. The exclusion criteria were: 1) patients admitted for recurrence of the same disease within 4 weeks; 2) patients with immunodeficiency; 3) patients with rheumatic disease, autoimmune disease, or malignant disease; 4) patients receiving immunosuppressive agents; and 5) patients moved from other medical areas within 2 weeks before admissions 1).

The diagnosis of KD was made in accordance with the Japanese diagnostic guidelines for KD.[1] Both complete and incomplete KD were included. The included infectious diseases were: respiratory tract infections (e.g., respiratory syncytial virus [RSV], human metapneumovirus [hMPV], adenovirus, influenza

© 2021 Hara T et al. *JAMA Network Open.*

virus, *Streptococcus pyogenes*], gastrointestinal infections (e.g., rotavirus, norovirus), exanthema subitum, and skin and soft tissue infections. In patients with respiratory tract infections or gastrointestinal infections, the pathogens were identified by serological tests, culture, or rapid immunochromatographic tests.

To investigate the nationwide trend, we performed an analysis using KD data from the KD Rapid Report System, the report system for selected institutions in Japan (http://www.kawasaki-disease.net/kawasakidata/). We used the data of 2,161 patients with KD from 11 hospitals in other areas of Japan that consistently reported exact numbers of patients with KD year-round from 2017–2020. To determine the ratio of KD to a specific infectious disease, we used the number of patients with RSV or rotavirus infection, obtained from the nationwide sentinel system incorporating weekly updated information from 3,000 pediatric institutions in Japan, National Epidemiological Surveillance for Infectious Diseases (NESID; https://www.niid.go.jp/niid/ja/ idwr.html).

eAppendix 2. Data Collection

The clinical information, including days of illness on admission and at diagnosis, history, clinical manifestations (duration of fever, other symptoms), laboratory data, treatment, cardiac involvement, and prognosis, of patients with KD was reviewed. Laboratory data included the white blood cell count (WBC), neutrophil percentage, platelet count, and levels of hemoglobin, total bilirubin, aspartate transaminase, alanine aminotransferase, serum sodium, and C-reactive protein. Treatment details included the day of

© 2021 Hara T et al. *JAMA Network Open.*

illness and dose of first intravenous immunoglobulin (IVIG) treatment, other intensification therapy, and requirement for any additional therapy. Cardiac assessment included left ventricular function, coronary diameter, valvular regurgitation, and pericardial effusion, which were evaluated at three timepoints: pretreatment, within 4 weeks (worst data), and after 4 weeks. For KD patients admitted in 2020, SARS-CoV-2 RNA and anti-SARS-CoV-2 IgG antibodies were assessed by RT-PCR (CFX96 Touch Deep Well; Bio-Rad) and a serological test (Elecsys® Anti-SARS-CoV-2; Roche Diagnostics K.K.), respectively. IVIG resistance was defined as occurring when patients with KD required additional IVIG therapy after the first IVIG treatment. The presence of a coronary arterial lesion was defined by the Z-score for the luminal diameter of one coronary artery being >2.5. In patients with infectious diseases, the final diagnosis, diagnostic methods, and pathogens were reviewed to evaluate specific pathogens before, during, and after the COVID-19 State of Emergency.

eAppendix 3. Statistical Analysis

Statistical analyses were performed using R, version 3.6.3 (The R foundation) and JMP Pro, version 15 software (SAS Institute Inc.). Neutrophil percentage data for two patients and total bilirubin data for seven patients were missing, and one pre-treatment echocardiogram was not performed. Data are presented as the mean (standard deviation) or median (interquartile range) for continuous variables and as the count (percentage) for categorical variables. Continuous variables were tested for normalcy using a Shapiro–Wilk

© 2021 Hara T et al. *JAMA Network Open.*

test and compared between groups using an unpaired Student's *t*-test or the Mann–Whitney U test, as appropriate. Categorical variables were compared using Fisher's exact test. The incidences of KD and infectious diseases were analyzed using a Poisson regression model. To account for possible over dispersion, the scale parameter was estimated by the square root of Pearson's Chi-Square divided by the degree of freedom. A two-tailed *P*-value of <0.05 was chosen as the cutoff for significance.

eReference

1. Ayusawa M, Sonobe T, Uemura S, et al. Revision of diagnostic guidelines for Kawasaki disease (the 5th revised edition). *Pediatr Int.* 2005;47(2):232-234.

© 2021 Hara T et al. *JAMA Network Open.*

eFigure 1. Geographical Locations of the FCH KD Center and Five Adjacent Hospitals, and the Number of Patients With KD

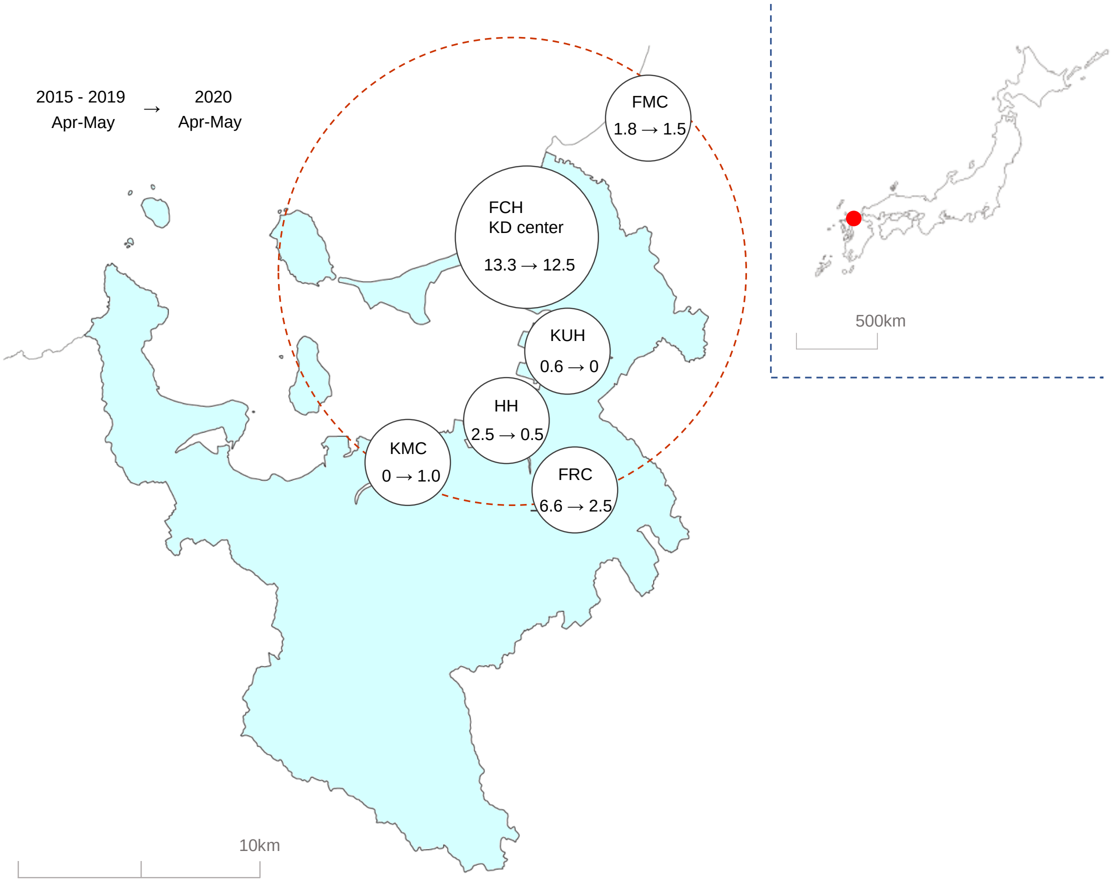

The light blue area shows Fukuoka city, and the six circles indicate the hospitals that participated in this research. The numbers of patients with KD in April–May, 2015–2019 and 2020 in each hospital are shown within each circle.

Institutions: Fukuoka Children's Hospital (FCH), Kyushu University Hospital (KUH), National Hospital Organization Kyushu Medical Center (KMC), Japanese Red Cross Fukuoka Hospital (FRC), Hamanomachi Hospital (HH), National Hospital Organization Fukuokahigashi Medical Center (FMC).

KD: Kawasaki disease

© 2021 Hara T et al. *JAMA Network Open.*

eFigure 2. Ratios of the Number of Patients With KD to That of Patients With RSV or Rotavirus Infection Across Six Hospitals in Fukuoka

A: The ratio of KD to RSV infection

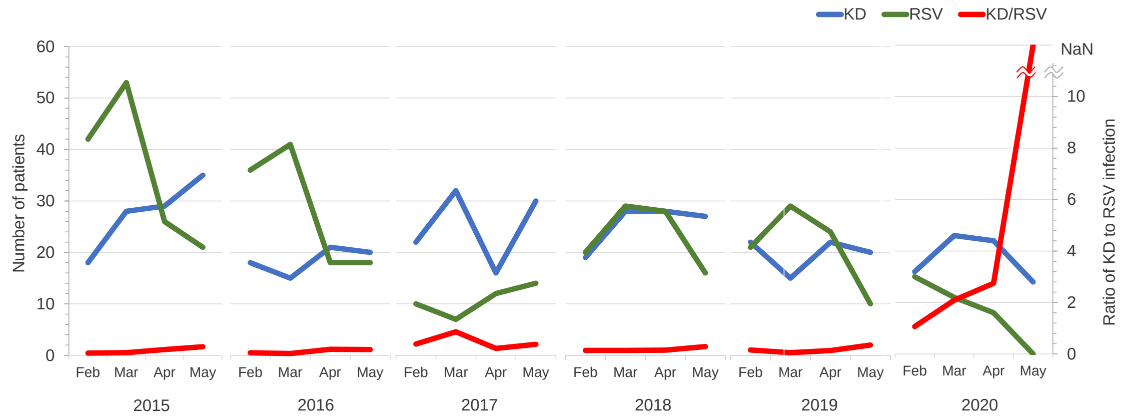

B. The ratio of KD to rotavirus infection

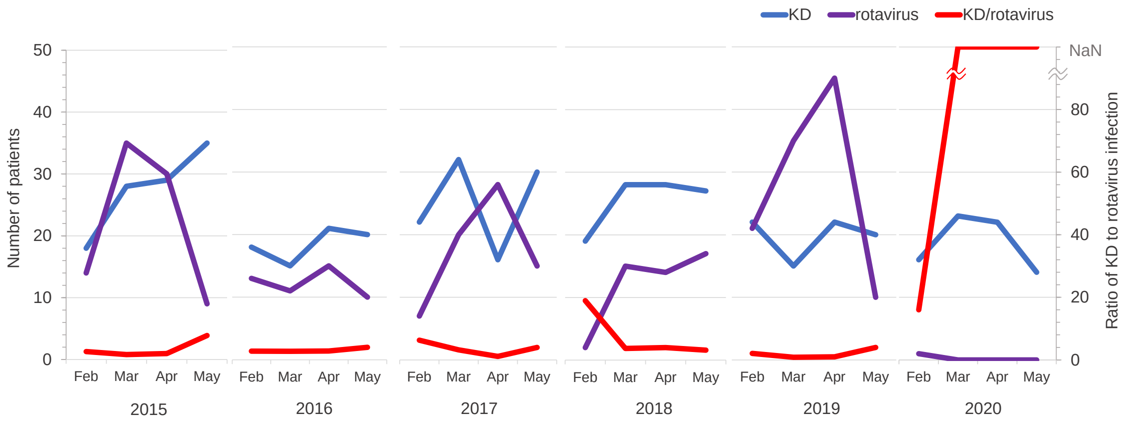

The red line represents the ratios of the number of patients with KD to that of patients with RSV or rotavirus infection. The blue line represents the number of patients with KD. The green and purple lines represent the numbers of patients with RSV and rotavirus infections, respectively.

KD: Kawasaki disease, RSV: respiratory syncytial virus, NaN: Not a Number, which means the number was divided by zero.

© 2021 Hara T et al. *JAMA Network Open.*

eFigure 3. Timeline of COVID-19 Cases and Policies in Japan

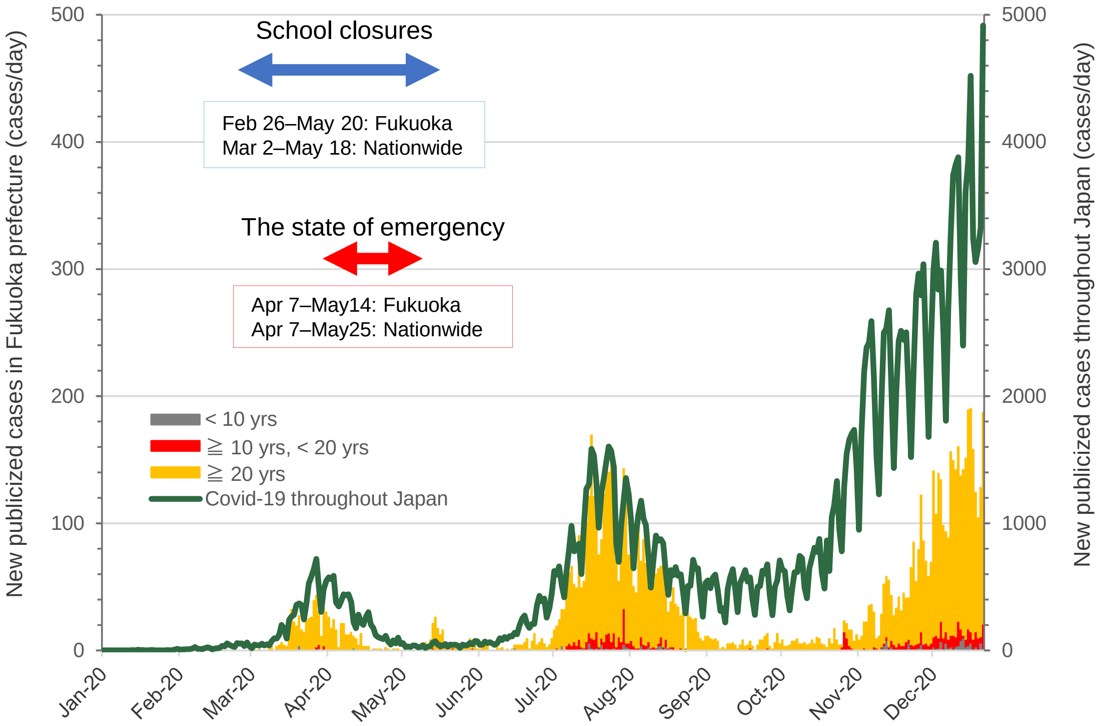

Bars represent the number of COVID-19 cases in Fukuoka prefecture, where gray, red, and yellow represent cases in patients aged less than 10 years, from 10 to less than 20 years, and greater than 20 years, respectively. The green line indicates the number of COVID-19 cases throughout Japan (right vertical axis). At the time of writing, there were 3,159 and 7,379 cases in patients aged less than 10 years and in those aged from 10 to less than 20 years, respectively, reported throughout Japan from January to November.

COVID-19: coronavirus disease 2019

© 2021 Hara T et al. *JAMA Network Open.*
